# SARS‐CoV‐2 Infection Aggravates Physical and Mental Health in Cancer Patients Compared to Co‐Living Individuals

**DOI:** 10.1002/cam4.70795

**Published:** 2025-03-25

**Authors:** Jiayao Liu, Na Li, Bin Wang, Wujie Zhao, Jie Zhi, Xiaojing Jia, Yitao Jia, Yanqing Tie

**Affiliations:** ^1^ The Third Department of Oncology Hebei General Hospital Shijiazhuang China; ^2^ Hebei Medical University Shijiazhuang China; ^3^ Department of Clinical Laboratory Hebei General Hospital Shijiazhuang China

**Keywords:** anxiety, cancer, co‐living individuals, COVID‐19, depression, mental

## Abstract

**Introduction:**

Cancer patients are particularly vulnerable to the psychological sequels of COVID‐19 due to their immunocompromised state and the disruptions to their regular oncological care. There is limited research comparing the effects of SARS‐CoV‐2 on cancer patients and their co‐living individuals. This study aims to explore the similarities and differences in physical and psychological outcomes between these two groups, with a 1‐year follow‐up to assess long‐term effects.

**Methods:**

This retrospective observational study was conducted between January and February 2023. A total of 107 participants were included: 72 cancer patients and 35 co‐living individuals, all diagnosed with COVID‐19. Clinical and laboratory data were collected. Depression, anxiety, and fatigue were assessed at two timepoints: shortly after COVID‐19 diagnosis and 1 year later.

**Results:**

Cancer patients exhibited higher rates of gastrointestinal symptoms, such as diarrhea (20.83% vs. 5.71%, *p* = 0.045), which were associated with increased anxiety and depression (*p* < 0.05). Advanced‐stage cancer (*p* < 0.01) and lack of vaccination (*p* < 0.01) correlated with worse psychological outcomes. Female cancer patients reported higher depression scores (*p* < 0.05). Laboratory findings indicated higher neutrophil percentages (*p* < 0.001), fibrinogen (*p* < 0.001), and D‐dimer levels (*p* = 0.015) in cancer patients, signaling a higher risk of inflammation and thrombosis. Both groups showed improvements in depression and fatigue over the 1‐year follow‐up, but cancer patients continued to report greater psychological distress (*p* < 0.001) and fatigue (*p* = 0.024).

**Conclusion:**

Cancer patients infected with COVID‐19 experienced more severe physical and psychological symptoms compared to their co‐living individuals, with persistent differences 1 year after infection.

**Trial Registration:**

ChiCTR2300067577

## 1 Introduction

Since its emergence in November 2019, COVID‐19, caused by the severe acute respiratory syndrome coronavirus 2 (SARS‐CoV‐2), has undergone significant evolution, with many variants being identified over time [1]. One such variant, Omicron, was first reported in South Africa in November 2021 and was quickly classified as a variant of concern by the World Health Organization (WHO) on November 26, 2021 [2]. Despite the WHO's declaration that COVID‐19 is no longer a global public health emergency, SARS‐CoV‐2 continues to pose a persistent health threat, particularly among vulnerable populations such as cancer patients.

Emerging evidence suggests that SARS‐CoV‐2 can persist long after initial infection. During the pandemic, cancer patients, already a vulnerable population, were shown to be at increased risk of anxiety and depression, particularly when delays in regular treatment were experienced [3, 4]. Beyond respiratory complications, COVID‐19 is also associated with gastrointestinal symptoms such as anorexia, nausea, vomiting, diarrhea, and abdominal pain [5, 6].

Cancer patients are at an increased risk of severe complications from COVID‐19 due to their immunocompromised state, whether from the malignancy itself or from cancer treatments such as chemotherapy, radiotherapy, or immunotherapy [7]. Additionally, the pandemic significantly disrupted healthcare access for these patients, leading to delayed treatments, reduced in‐person consultations, and increased psychological distress due to uncertainty about disease progression and prognosis [8]. Beyond the physiological impact, cancer patients face a heightened psychological burden, which may be exacerbated by disruptions in their treatment schedules, fear of disease progression, and isolation during the pandemic [9]. The extent of this burden may vary based on cancer characteristics, including tumor type, location, and stage, as more advanced disease, aggressive malignancies, or cancers affecting vital organs tend to be associated with greater emotional distress and fatigue. Several studies have documented increased rates of anxiety and depression in cancer patients during the COVID‐19 pandemic [10, 11]. However, less attention has been given to the psychological burden carried by their co‐living individuals (e.g., spouses or caregivers), who often experience emotional distress due to caregiving responsibilities and concern for their loved ones' health.

While prior studies have focused on the mental health burden of COVID‐19 in cancer patients, fewer have investigated how psychological distress extends to their co‐living individuals—such as spouses and caregivers [12, 13]. These individuals play a crucial role in providing emotional and physical support to cancer patients, often at the expense of their own well‐being. A study conducted in Singapore found that 72.8% of caregivers reported high levels of fear related to COVID‐19, with 22.5% experiencing clinically significant anxiety [13]. Similarly, research on parents of pediatric cancer patients during the pandemic revealed elevated post‐traumatic stress symptoms and high levels of perceived stress and anxiety, highlighting the extended psychological toll of COVID‐19 beyond the patients themselves [12]. The psychological burden on caregivers may be exacerbated by factors such as caregiving stress, fear of transmitting infection, and witnessing the deterioration of a loved one's health [14]. However, there remains a significant gap in understanding how COVID‐19 affects both cancer patients and their co‐living individuals over time, particularly regarding depression, anxiety, and fatigue.

This study aims to fill this gap by comparing the physical and psychological effects of SARS‐CoV‐2 infection on cancer patients and their co‐living individuals. Specifically, it evaluates the progression of anxiety, depression, and fatigue at two time points: shortly after infection and 1 year later. By identifying key differences and correlations between these psychological factors and clinical characteristics, this study seeks to provide valuable insights that can guide clinicians in the management and mental health support of cancer patients and their caregivers during and beyond the pandemic.

## 2 Materials and Methods

### 2.1 Study Design

The study population comprised 107 participants, including 72 cancer patients and 35 co‐living individuals, all of whom were diagnosed with COVID‐19. Recruitment occurred between January 3, 2023 and February 6, 2023. Comprehensive clinical and laboratory data were collected, including full blood counts, liver function tests, renal function tests, coagulation profiles, and immunoprotein measurements. The study adhered to the ethical guidelines outlined by the Declaration of Helsinki, and all authors had full access to the data to ensure integrity in reporting the findings.

### 2.2 Inclusion Criteria

Two distinct populations were included: cancer patients and their co‐living individuals (spouses or caregivers). The inclusion criteria were as follows:

Cancer patients:

- Histopathologically confirmed diagnosis of cancer (regardless of cancer type).
- Diagnosis of COVID‐19, confirmed using criteria established by the National Health Commission of the People's Republic of China (as per the “Pneumonia Treatment Protocol for Novel Coronavirus Infection, Trial Version 10”) [15].
- Infection confirmed to be caused by the Omicron variant of SARS‐CoV‐2. Omicron variant infection was confirmed either through whole‐genome sequencing of the SARS‐CoV‐2 virus or inferred based on variant‐specific PCR assays where sequencing was unavailable. In cases where genomic confirmation was not performed, the diagnosis was inferred epidemiologically, as the study period coincided with the dominance of the Omicron variant in the region.

Co‐living individuals:

- Spouses or caregivers cohabitating with cancer patients.
- Diagnosis of COVID‐19, confirmed using the same national criteria applied to cancer patients.
- Infection confirmed to be caused by the Omicron variant of SARS‐CoV‐2.

### 2.3 Exclusion Criteria

Participants were excluded if they met any of the following criteria:

Cancer patients:

- No evidence of SARS‐CoV‐2 infection.
- History of severe primary diseases affecting the liver, kidneys, hematopoietic system, or nervous system.
- History of heart failure or arrhythmia.
- Presence of severe mental disorders requiring constraint or intensive management.

Co‐living individuals:

- Presence of a malignant tumor.
- No evidence of SARS‐CoV‐2 infection.
- History of severe primary diseases affecting the liver, kidneys, hematopoietic system, or nervous system.
- History of heart failure or arrhythmia.
- Presence of severe mental disorders requiring constraint or intensive management.

### 2.4 Psychological Assessment

The primary endpoint of this study was to assess the long‐term psychological impact of COVID‐19 on cancer patients and their co‐living individuals, as measured by: depression severity (through Patient Health Questionnaire‐9 (PHQ‐9)), anxiety levels (through Generalized Anxiety Disorder‐7 (GAD‐7)), and fatigue burden (through the Fatigue Scale‐14 (FS‐14)) as follows:

- Depression: The PHQ‐9 is a 9‐item scale that assesses depressive symptoms over the past 2 weeks [16]. Each item is scored on a scale from 0 to 3 (0 = “not at all” to 3 = “nearly every day”), resulting in a total score ranging from 0 to 27 (a score of 10 is usually used to define clinically significant depression). A higher score indicates a greater depression severity.
- Anxiety: The GAD‐7 is a 7‐item scale that evaluates anxiety symptoms over the past 2 weeks [17]. Each item is rated on a scale from 0 to 3 (0 = “not at all” to 3 = “nearly every day”), with a total score ranging from 0 to 21 (a score of 10 or higher is used to indicate clinically significant anxiety). A higher score indicates greater degree of anxiety.
- Fatigue: The FS‐14 is a comprehensive 14‐item scale designed to assess both physical and mental fatigue [18]. Items 1–8 assess physical fatigue, and items 9–14 assess mental fatigue. Each item is scored from 0 to 3, and the total score ranges from 0 to 42, with higher scores indicating greater levels of fatigue. The Chinese version of the FS‐14 has been validated and found to have acceptable psychometric properties in assessing fatigue levels in various populations.

Psychological assessments were conducted twice: shortly after the diagnosis of COVID‐19 and 1 year later. Psychological data for the remaining participants were collected through telephone interviews or face‐to‐face interviews.

Secondary (exploratory) analyses included: subgroup comparisons (based on age, gender, cancer type, cancer stage, and presentation severity at COVID‐19 diagnosis), correlation analysis (between PHQ‐9, GAD‐7, and FS‐14 scores), and the differences between cancer patients and their co‐living individuals in psychological burden over time.

### 2.5 Data Collection and Management

Clinical data were collected from participants' medical records via the hospital's electronic medical record system. Data collected included demographic characteristics (e.g., age and sex), underlying health conditions, post‐COVID‐19 symptoms, and vaccination status. Laboratory data, including full blood counts, liver and renal function tests, coagulation profiles, and immunoprotein levels, were obtained from routine hospital diagnostic procedures.

Psychological assessments (PHQ‐9, GAD‐7, and FS‐14) were administered either by telephone or in person. Two researchers independently entered all data into Excel 2013 for data management and validation. The entries were cross‐checked to ensure data integrity, and discrepancies were resolved through re‐examination of the original records. Follow‐up assessments were conducted 1‐year post‐COVID‐19 infection to assess long‐term changes in psychological status.

### 2.6 Statistical Analysis

All statistical analyses were conducted using IBM SPSS Statistics software (version 25.0, IBM Corp). Continuous variables were expressed as the mean ± standard deviation (SD) or median and 95% confidence interval (CI), depending on the distribution of the data. For comparisons between groups, independent sample *t*‐tests were used for normally distributed variables with equal variance, while the Mann–Whitney *U* test was applied for non‐normally distributed data. Categorical variables were analyzed using chi‐square tests.

Longitudinal changes in psychological measures (PHQ‐9, GAD‐7, and FS‐14) between baseline and 1 year after COVID‐19 infection were evaluated using the Wilcoxon signed‐rank test. Correlations between psychological scores and other clinical variables were assessed using Spearman's rank correlation coefficient. Analysis of variance (ANOVA) was employed to compare continuous variables across subgroups where appropriate. Statistical significance was set at *p* < 0.05 for all analyses.

## 3 Results

### 3.1 Baseline and Demographic Characteristics

**FIGURE 1:**
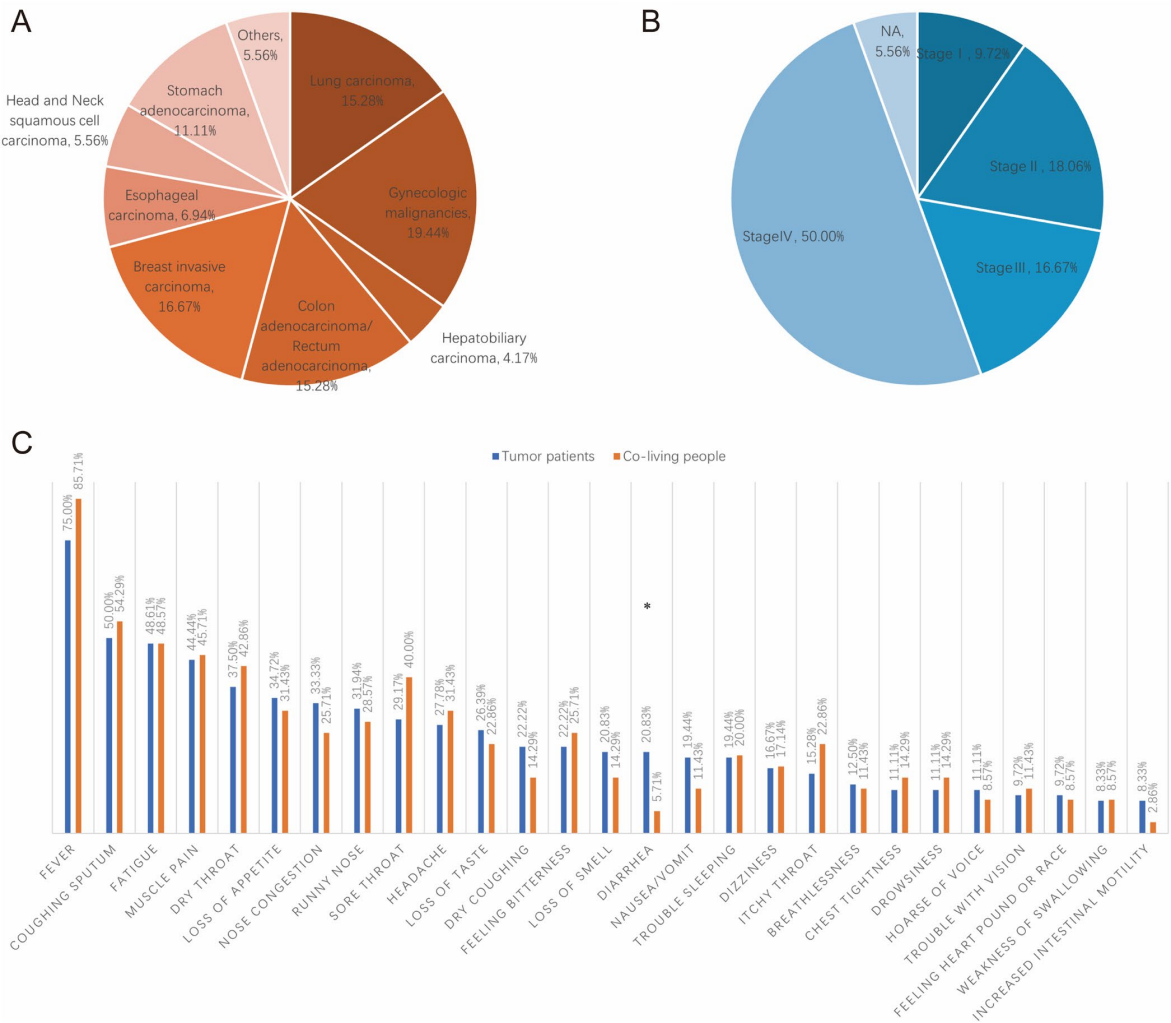
An illustration showing the distribution of cancer patients based on tumor type (A), stage (B), and presenting symptoms in comparison with co‐living individuals (C).

During the 1‐year follow‐up, 16 cancer patients had died, two cancer patients were lost to follow‐up, and seven co‐living individuals were lost to follow‐up. A total of 107 participants were included in the study, comprising 72 cancer patients and 35 co‐living individuals. The demographic characteristics between the two groups showed significant differences in terms of sex distribution (Table 1). Among the cancer patients, 31.94% were male and 68.06% were female, while in the co‐living group, 65.71% were male and 34.29% were female, which was a statistically significant difference (*p* = 0.001). However, the age distribution between the groups was not significantly different (*p* = 0.611). The majority of cancer patients (43.06%) and co‐living individuals (57.14%) were 61 years or older, with the remainder spread across younger age categories. In patients with cancer, gynecologic malignancy was the most common type (19.44%) followed by invasive carcinoma of the breast (16.67%), lung cancer (15.28%), and colorectal adenocarcinoma (15.28%), respectively (Figure 1A). In terms of tumor staging, the majority of cases had stage IV tumors (50%) followed by stage II (18.06%) and stage III (16.67%), respectively (Figure 1B).

**TABLE 1:** General characteristics and demographics of the patients and co‐living individuals.

Variable	Cancer patients	Co‐living individuals	χ^2^	*p*
	(*n* = 72)	(*n* = 35)		
Sex				
Male	23 (31.94%)	23 (65.71%)	10.959	**0.001**
Female	49 (68.06%)	12 (34.29%)		
Age				
≤ 40 years	7 (9.72%)	2 (5.71%)	1.922	0.611
41–50 years	10 (13.89%)	3 (8.57%)		
51‐60 years	24 (33.33%)	10 (28.57%)		
≥ 61 years	31 (43.06%)	20 (57.14%)		
Vaccination status of COVID‐19				
Unvaccinated	25 (34.72%)	2 (5.71%)	10.504	**0.001**
Vaccinated	47 (65.28%)	33 (94.29%)		
Numbers of post‐infection symptoms				
< 5	29 (40.28%)	15 (42.86%)	1.801	0.406
10‐May	27 (37.5%)	9 (25.71%)		
> 10	16 (22.22%)	11 (31.43%)		
Initial symptoms				
Fever	51 (75%)	30 (85.71%)	7.071	0.259
Cough	7 (7.72%)	1 (2.86%)		
Sore throat	1 (1.39%)	2 (5.71%)		
Muscle aches	4 (5.56%)	0 (0%)		
Fatigue	6 (8.33%)	1 (2.86%)		
Headache	2 (2.78%)	0 (0%)		
Asymptomatic	1 (1.39%)	1 (2.86%)		
Severity of fever				
≤ 38.0°C	24 (45.28%)	12 (40%)	1.352	0.515
> 38.0°C–39.0°C	22 (41.51%)	16 (53.33%)		
≥ 39.1°C	7 (13.21%)	2 (6.67%)		

	Cancer patients	Co‐living individuals	F/Z	*p*
Laboratory data				
White blood cell count (×10^9^/L)	5.66 (3.343)	5.59 (1.513)	0.017	0.895
Neutrophils (%)	68.28 (9.636)	53.76 (7.439)	61.466	**< 0.001**
Lymphocytes (%)	22.93 (8.239)	36.38 (7.068)	68.64	**< 0.001**
Red blood cell count (×10^12^/L)	3.99 (0.624)	4.47 (0.520)	15.586	**< 0.001**
Hemoglobin (g/L)	121.15 (16.318)	136.60 (15.849)	21.5	**< 0.001**
Platelet count (×10^9^/L)	239.00 (94.359)	253.91 (78.742)	0.653	0.27
Prothrombin time (s)	11.51 (0.773)	11.37 (0.589)	0.975	0.326
Activated partial thromboplastin time (s)	26.38 (2.134)	26.31 (1.916)	0.028	0.868
Fibrinogen contents (g/L)	3.52 (1.097)	2.75 (0.481)	15.848	**< 0.001**
D‐dimer (mg/L)	0.83 (1.117)	0.42 (0.403)	2.43	**0.015**
Total protein (g/L)	71.62 (7.465)	68.41 (5.988)	4.917	**0.029**
Albumin (g/L)	40.52 (4.650)	41.65 (3.378)	1.656	0.201
Prealbumin (mg/dL)	20.01 (5.864)	21.26 (4.368)	1.248	0.266
Alanine aminotransferase (U/L)	21.56 (18.268)	23.08 (21.093)	0.495	0.621
Aspartate aminotransferase (U/L)	26.00 (14.192)	24.01 (10.179)	1.215	0.224
AST/ALT (1.15)	1.56 (0.867)	1.33 (0.650)	1.584	0.113
Urea nitrogen (mmol/L)	4.67 (1.385)	5.23 (1.248)	4.105	**0.045**
Creatinine (μmol/L)	64.77 (24.858)	67.13 (13.728)	0.275	0.601
C‐reactive protein (mg/L)	11.80 (30.584)	1.46 (1.586)	1.874	0.061
Immunoglobulin G (g/L)	12.19 (3.058)	11.39 (2.811)	1.716	0.193
Immunoglobulin A (g/L)	2.35 (0.894)	2.18 (1.027)	0.781	0.379
Immunoglobulin M (g/L)	1.17 (0.579)	0.78 (0.328)	14.15	**< 0.001**
Complements C3 (g/L)	1.26 (0.292)	1.12 (0.168)	6.753	**0.011**
Complements C4 (g/L)	0.24 (0.080)	0.22 (0.056)	1.232	0.218
Complements C1q (mg/L)	170.87 (29.319)	158.89 (24.430)	4.7	**0.032**

*Note:* Bolded values indicate statistically significant findings (*p*<0.05).

Abbreviations: ALT, alanine aminotransferase; AST, aspartate aminotransferase.

COVID‐19 vaccination was significantly higher among co‐living individuals compared to cancer patients (94.29% vs. 65.28%, *p* = 0.001). Overall, 27 different symptoms were reported, of which fever was the most common symptom in both cancer patients and co‐living individuals (75% vs. 85.71%) followed by productive cough (50% vs. 54.29%), fatigue (48.6% vs. 48.57%), and muscle pain (44.44% vs. 45.71%), respectively. No significant difference was noted between both groups in terms of fever severity (*p* = 0.515) (Figure 1C). Most cancer patients and co‐living individuals had less than five post‐COVID‐19 infection symptoms (40.28% vs. 42.86%).

Laboratory analyses revealed several significant differences between cancer patients and co‐living individuals (Table 1). The neutrophil percentage was significantly higher in cancer patients compared to co‐living individuals (68.28 vs. 53.76), with a highly significant difference (*p* < 0.001). Lymphocyte percentages, on the other hand, were notably lower in cancer patients compared to co‐living individuals (22.93 vs. 36.38, *p* < 0.001). In terms of red blood cell count, cancer patients had significantly lower levels compared to co‐living individuals (3.99 vs. 4.4710^12^/L), with a highly significant difference (*p* < 0.001). Hemoglobin levels followed a similar trend, with cancer patients showing significantly lower levels compared to co‐living individuals (121.15 vs. 136.60 g/L, *p* < 0.001).

The fibrinogen levels were significantly higher in cancer patients compared to co‐living individuals (3.52 vs. 2.75 g/L, *p* < 0.001). Similarly, D‐dimer levels were significantly elevated in cancer patients compared to co‐living individuals (0.83 vs. 0.42 mg/L, *p* = 0.015). Total protein levels were slightly higher in cancer patients compared to co‐living individuals (71.62 vs. 68.41 g/L, *p* = 0.029), and urea nitrogen levels were significantly lower in cancer patients compared to co‐living individuals (4.67 vs. 5.23 mmol/L, *p* = 0.045). Complement levels also varied between the two groups. Cancer patients exhibited significantly higher levels of complement C3 (1.26 ± 0.292 g/L) compared to co‐living individuals (1.26 vs. 1.12 g/L, *p* = 0.011), and complement C1q levels were also elevated in cancer patients (170.87 ± 29.319 mg/L) compared to co‐living individuals (170.87 vs. 158.89 mg/L, *p* = 0.032). There were no significant differences in white blood cell count, platelet count, prothrombin time, activated partial thromboplastin time, or several liver function‐related indicators such as alanine aminotransferase (ALT) and aspartate aminotransferase (AST) between the two groups.

### 3.2 FS‐14, PHQ‐9, and GAD‐7 Scores Between Cancer Patients and Co‐Living Individuals

The changes in depression, anxiety, and fatigue between examined groups and at different assessment timepoints are presented in Table 2. The analysis of mean changes in psychological scores from baseline to 1‐year post‐COVID‐19 showed a significant reduction in depression for both cancer patients (mean difference: −1.55, *p* = 0.045) and co‐living individuals (mean difference: −1.49, *p* = 0.004). However, the magnitude of reduction was comparable between the two groups. Anxiety scores decreased in both groups but did not reach statistical significance (*p* = 0.277 for cancer patients, *p* = 0.416 for co‐living individuals). Fatigue levels significantly declined over 1 year for both cancer patients (mean difference: −2.55, *p* < 0.001) and co‐living individuals (mean difference: −2.66, *p* = 0.002), with a slightly greater reduction observed in co‐living individuals (*p* = 0.024 for intergroup comparison).

**TABLE 2:** Depression, anxiety, and fatigue scores of cancer patients and their co‐living individuals.

		Baseline (just after contracting COVID‐19)	1 year after COVID‐19	*Z*	*p*	Mean difference
PHQ‐9	Cancer patients	4.11 (3.91)	2.56 (2.51)	2.005	**0.045**	−1.55
	Co‐living individuals	2.20 (2.56)	0.71 (1.01)	2.845	**0.004**	−1.49
	*Z*	2.609	4.154	—	—	—
	*p*	**0.009**	**< 0.001**	—	—	—
GAD‐7	Cancer patients	2.86 (4.30)	2.11 (2.55)	1.087	0.277	−0.75
	Co‐living individuals	1.03 (2.18)	0.46 (0.69)	0.814	0.416	−0.57
	*Z*	2.835	3.476	—	—	—
	*p*	**0.005**	**0.001**	—	—	—
FS‐14	Cancer patients	5.61 (3.27)	3.06 (2.63)	4.455	**< 0.001**	−2.55
	Co‐living individuals	4.37 (3.46)	1.71 (1.78)	3.120	**0.002**	−2.66
	*Z*	1.824	2.255	—	—	—
	*p*	0.068	**0.024**	—	—	—

*Note:* Bolded values indicate statistically significant findings (*p*<0.05).

Abbreviations: FS‐14: 14‐item Fatigue scale; GAD‐7, general anxiety disorder‐7; *p*, *p* value; PHQ‐9, Patient Health Questionnaire‐9

### 3.3 The Association Between Psychological Parameters and Clinicodemographic Characteristics of Cancer Patients

**FIGURE 2:**
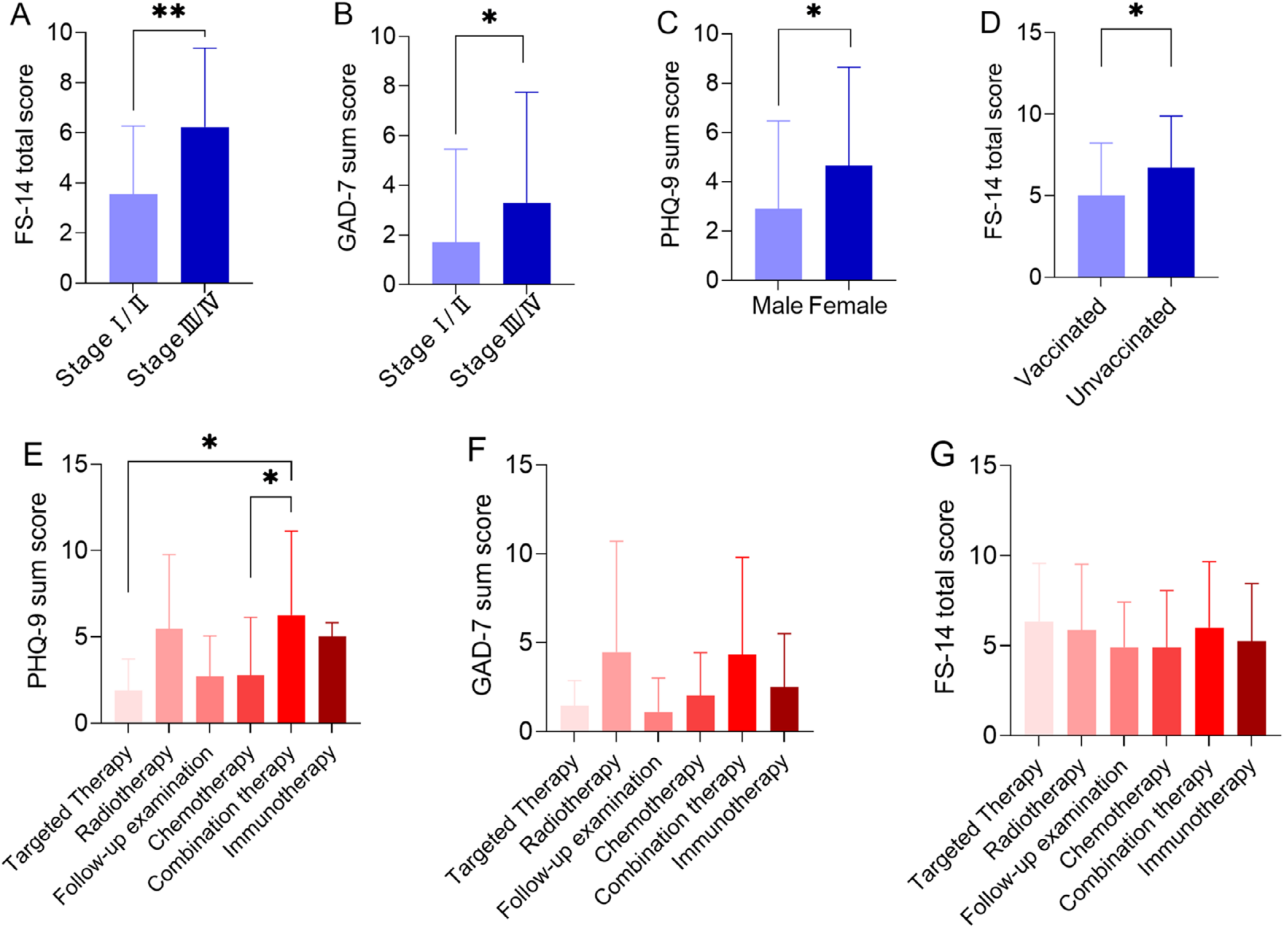
The differences in FS‐14, PHQ‐9, and GAD‐7 scores among cancer patients based on cancer stage (A, B), gender (C), vaccination status (D), and treatment modality (E–G). *p < 0.05; **p < 0.01. FS‐14, 14‐item Fatigue scale; GAD‐7, general anxiety disorder‐7; PHQ‐9, Patient Health Questionnaire‐9.

Figure 2 presents the differences in fatigue (FS‐14), depression (PHQ‐9), and anxiety (GAD‐7) scores among cancer patients, stratified by cancer stage, gender, vaccination status, and treatment modality.

Significant differences in fatigue and anxiety scores were observed based on cancer stage. Patients with advanced‐stage cancer (Stage III/IV) had significantly higher FS‐14 fatigue scores compared to those with early‐stage disease (Stage I/II) (*p* < 0.01) (Figure 2A). Similarly, patients with Stage III/IV cancer exhibited significantly higher GAD‐7 anxiety scores than those with Stage I/II disease (*p* < 0.05) (Figure 2B), indicating greater psychological distress in patients with more advanced disease.

A significant difference in depression scores was observed between male and female patients, with female patients reporting significantly higher depression levels compared to males (*p* < 0.05) (Figure 2C). This suggests that gender may play a role in the psychological impact of COVID‐19 on cancer patients, with females being more vulnerable to depressive symptoms.

Patients who were unvaccinated against COVID‐19 had significantly higher FS‐14 fatigue scores compared to those who were vaccinated (*p* < 0.05) (Figure 2D). This finding suggests that vaccination status may influence fatigue levels in cancer patients, with unvaccinated individuals experiencing more severe fatigue.

When stratified by treatment modality, significant differences in depression scores were observed (Figure 2E). Patients receiving combination therapy exhibited significantly higher PHQ‐9 scores compared to those undergoing chemotherapy (*p* < 0.05) and those receiving targeted therapy (*p* < 0.05), indicating a higher burden of depression in patients receiving more intensive treatment regimens. However, no significant differences were found in GAD‐7 anxiety scores (Figure 2F) or FS‐14 fatigue scores (Figure 2G) across different treatment modalities.

**FIGURE 3:**
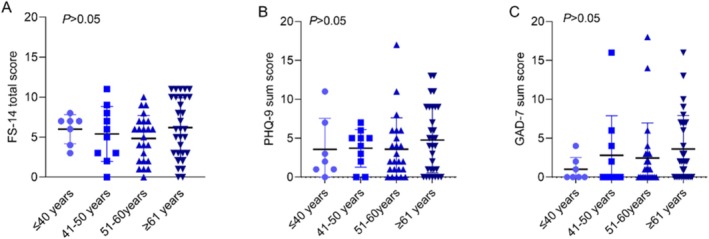
The differences in FS‐14, PHQ‐9, and GAD‐7 scores of cancer patients according to age. (A) FS‐14 score; (B) PHQ‐9 score; (C) GAD‐7 score. FS‐14, 14‐item Fatigue scale; GAD‐7, general anxiety disorder‐7; PHQ‐9, Patient Health Questionnaire‐9.

Although differences in fatigue, depression, and anxiety scores were noted in different cancer types (Table S1) and at different age groups (Figure 3); however, they did not reach a statistically significant level.

**FIGURE 4:**
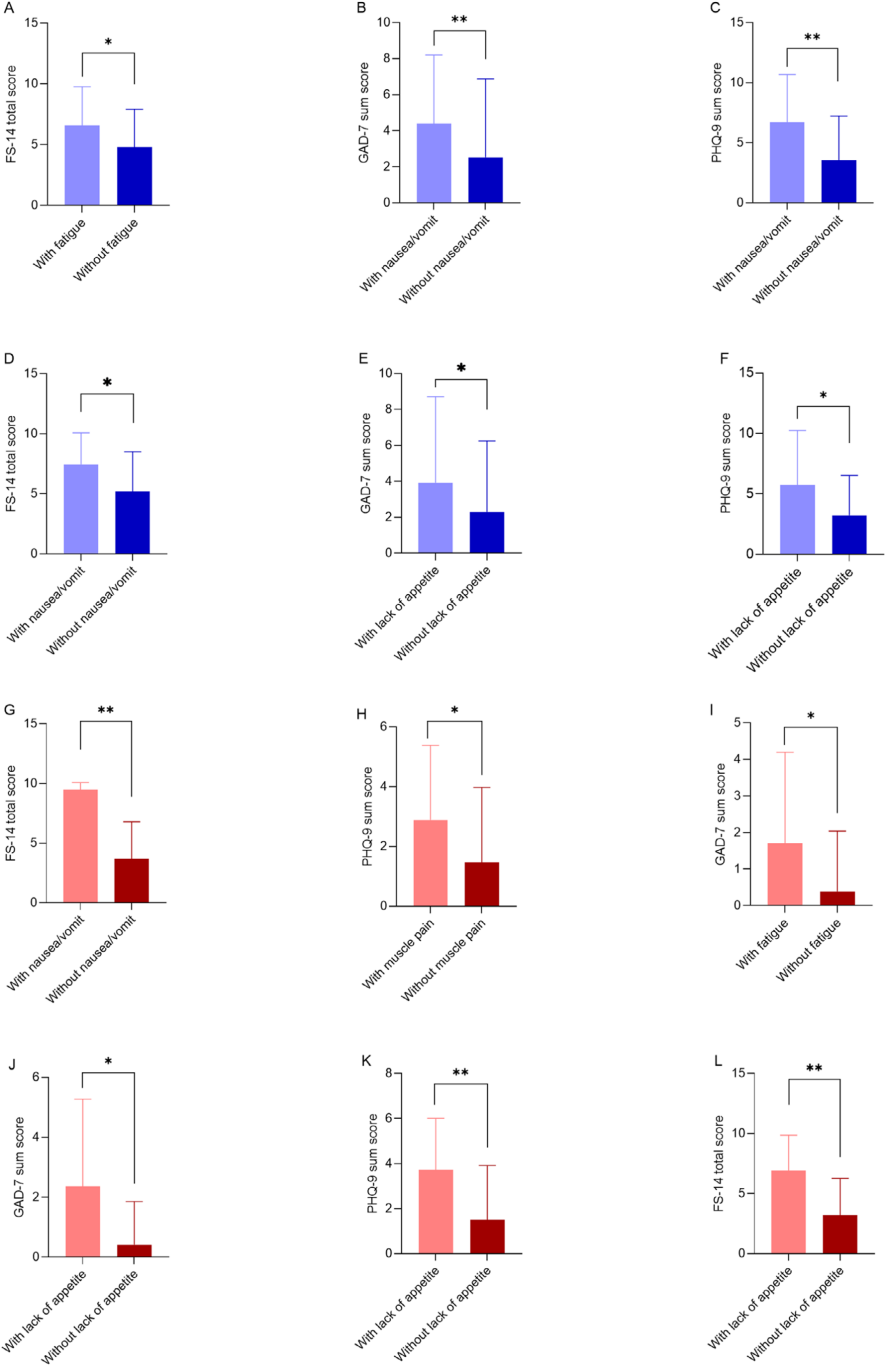
The association between FS‐14, PHQ‐9, and GAD‐7 scores and presenting symptoms among cancer patients (A–F) and co‐living individuals (G–L). FS‐14, 14‐item Fatigue scale; GAD‐7, general anxiety disorder‐7; PHQ‐9, Patient Health Questionnaire‐9. *p < 0.05; **p < 0.01.

The associations between different physical symptoms and FS‐14, PHQ‐9, and GAD‐7 scores in cancer patients are illustrated in Figure 4A–F. A significant association was observed between fatigue and FS‐14 scores, where patients reporting higher levels of fatigue had significantly elevated FS‐14 fatigue scores (*p* < 0.05). This indicates a robust correlation between subjective fatigue complaints and their measured fatigue scale. Furthermore, nausea and vomiting were significantly associated with increased GAD‐7 anxiety scores (*p* < 0.01), suggesting that these gastrointestinal symptoms contributed to heightened anxiety levels. A similar relationship was noted between nausea/vomiting and PHQ‐9 depression scores (*p* < 0.01), indicating that these physical symptoms were also linked to higher depressive symptoms. Additionally, nausea and vomiting were associated with significantly higher FS‐14 fatigue scores (*p* < 0.05), highlighting the impact of these gastrointestinal symptoms on both fatigue and psychological distress.

The lack of appetite in cancer patients was also significantly related to psychological symptoms. Specifically, patients experiencing a loss of appetite had significantly higher GAD‐7 anxiety scores (*p* < 0.05), demonstrating that appetite disturbances may exacerbate anxiety. Similarly, lack of appetite was associated with higher PHQ‐9 depression scores (*p* < 0.05), further emphasizing the link between physical symptoms and psychological distress in this patient group.

### 3.4 The Association Between Psychological Parameters and Baseline Clinicodemographic Characteristics of Co‐Living Individuals

The associations between different physical symptoms and FS‐14, PHQ‐9, and GAD‐7 scores in co‐living individuals are illustrated in Figure 4G–L. Nausea and vomiting were significantly associated with increased FS‐14 fatigue scores (*p* < 0.01), consistent with the findings in cancer patients. This suggests that these gastrointestinal symptoms contribute to fatigue in both groups. Muscle pain was another symptom associated with psychological distress, as co‐living individuals reporting muscle pain exhibited significantly higher PHQ‐9 depression scores (*p* < 0.05). Additionally, fatigue was significantly associated with increased GAD‐7 anxiety scores (*p* < 0.05), suggesting that physical exhaustion may exacerbate anxiety in co‐living individuals.

**FIGURE 5:**
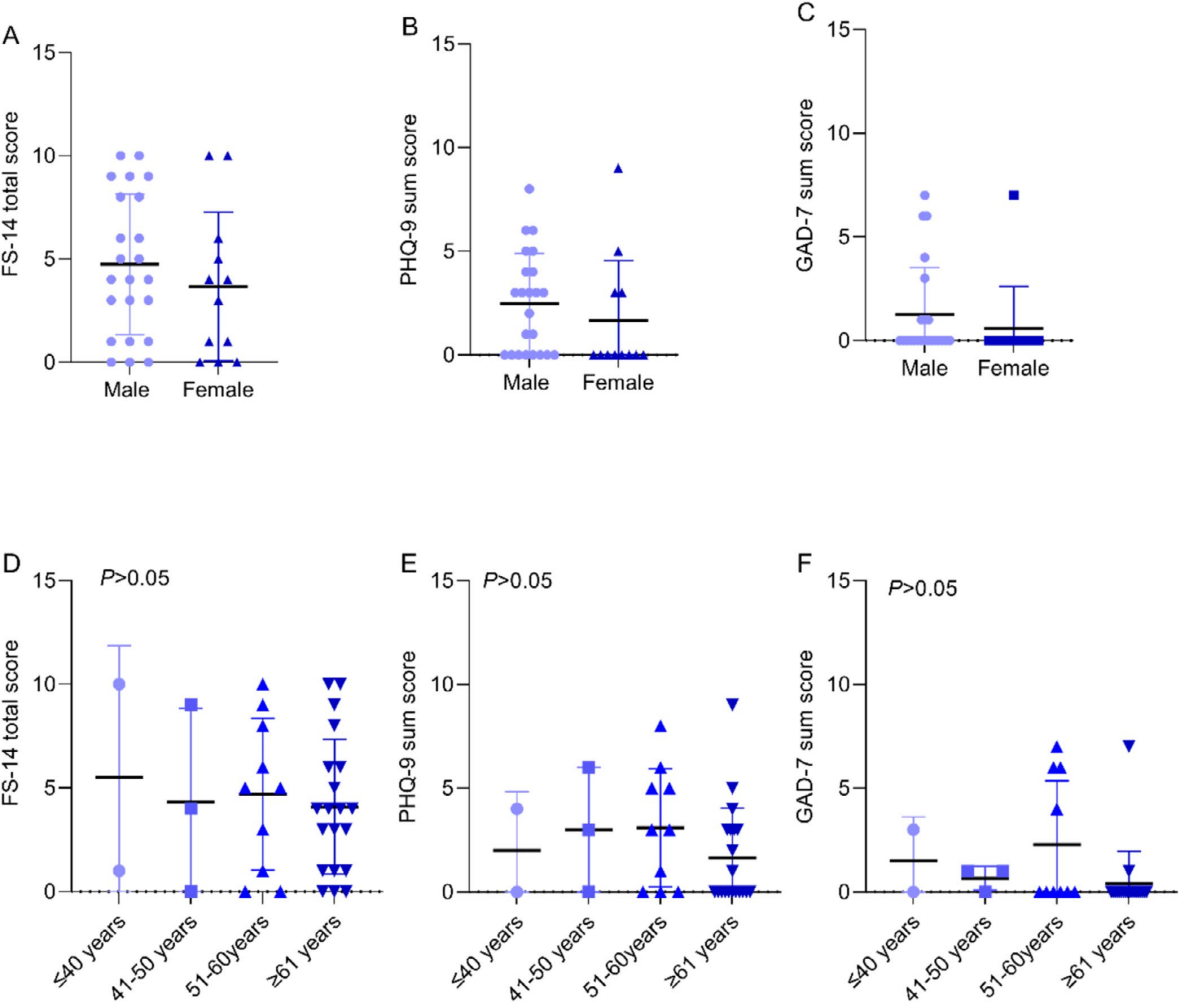
The differences in FS‐14, PHQ‐9, GAD‐7 scores of co‐living individuals based on gender (A–C) and age (D–F). FS‐14, 14‐item Fatigue scale; GAD‐7, general anxiety disorder‐7; PHQ‐9, Patient Health Questionnaire‐9.

Loss of appetite was significantly linked to psychological outcomes in co‐living individuals, similar to the cancer patient group. Specifically, a loss of appetite was associated with elevated GAD‐7 anxiety scores (*p* < 0.05) and increased PHQ‐9 depression scores (*p* < 0.01), indicating a strong relationship between appetite disturbances and psychological distress. Furthermore, co‐living individuals with a loss of appetite had significantly higher FS‐14 fatigue scores (*p* < 0.01), suggesting that appetite loss is also associated with increased fatigue in this population. Individuals' age and gender were not significantly correlated with the FS‐14, PHQ‐9, and GAD‐7scores (Figure 5).

### 3.5 The Correlation Between Psychological Scores (FS‐14, PHQ‐9, and GAD‐7) Among Cancer Patients and Co‐Living Individuals

**FIGURE 6:**
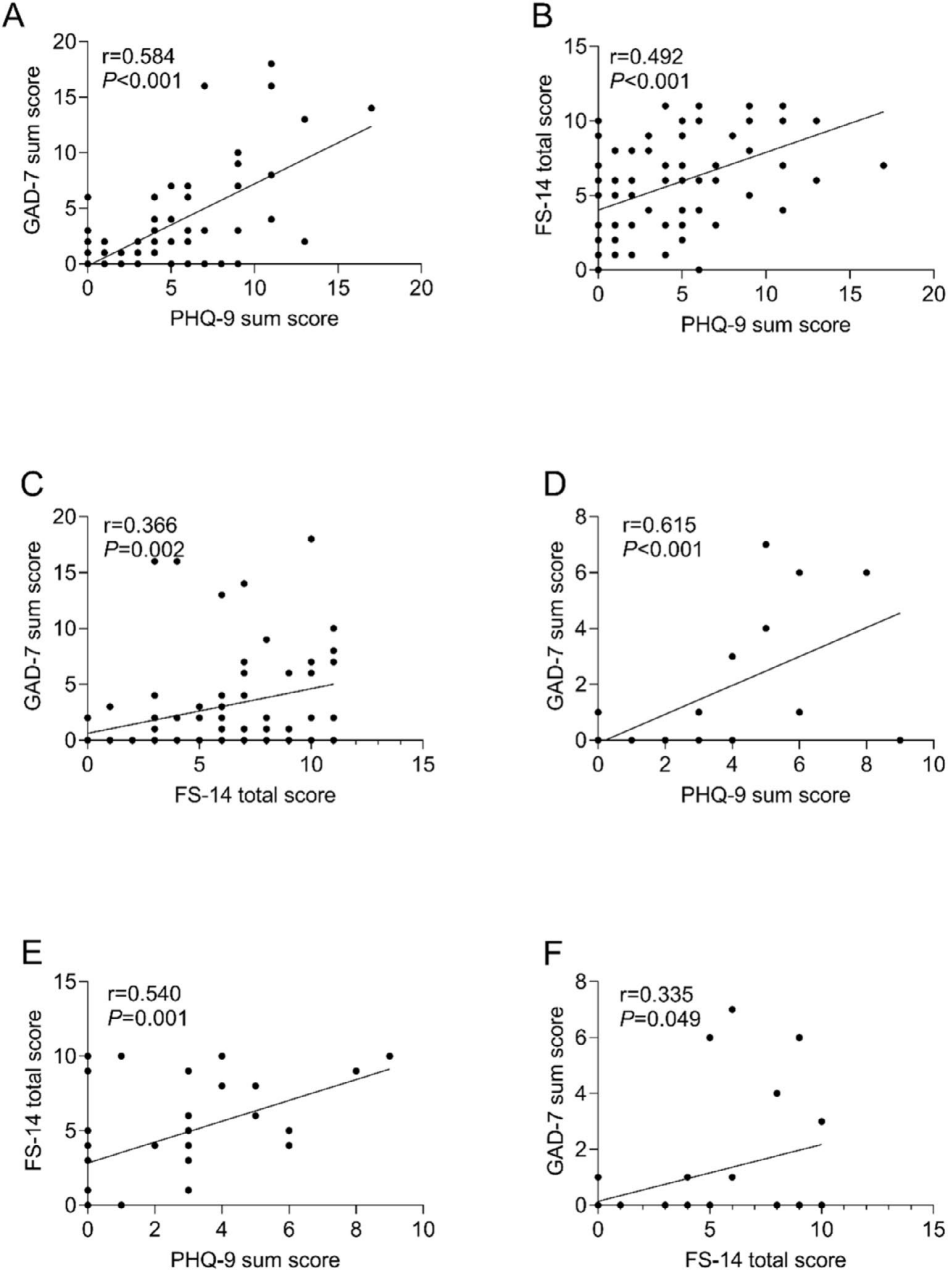
The correlations between the different psychological measures (FS‐14, PHQ‐9, and GAD‐7) both examined groups (A) PHQ‐9 and GAD‐7 in cancer patients; (B) PHQ‐9 and FS‐14 in cancer patients; (C) FS‐14 and GAD‐7 in cancer patients; (D) PHQ‐9 and GAD‐7 in co‐living individuals; (E) PHQ‐9 and FS‐14 in co‐living individuals; (F) FS‐14 and GAD‐7 in co‐living individuals.

Figure 6 illustrates the correlations between FS‐14, PHQ‐9, and GAD‐7 scores in both cancer patients and co‐living individuals. In the cancer patient group, a significant positive correlation was observed between PHQ‐9 and GAD‐7 scores (*r* = 0.584, *p* < 0.001), indicating that higher depression scores were associated with greater anxiety. Similarly, PHQ‐9 scores were significantly correlated with FS‐14 scores (*r* = 0.492, *p* < 0.001), suggesting that increased depression was associated with higher levels of fatigue in these patients. A moderate correlation was also found between FS‐14 and GAD‐7 scores (*r* = 0.366, *p* = 0.002), indicating that patients with greater fatigue tended to report higher anxiety levels.

In the co‐living group, a strong positive correlation was observed between PHQ‐9 and GAD‐7 scores (*r* = 0.615, *p* < 0.001), consistent with the findings in the tumor group. The correlation between PHQ‐9 and FS‐14 scores was also significant (*r* = 0.540, *p* = 0.001), indicating that higher depression scores were associated with greater fatigue. However, the correlation between FS‐14 and GAD‐7 scores was weaker but still significant (*r* = 0.335, *p* = 0.049), suggesting a modest relationship between fatigue and anxiety in co‐living individuals.

## 4 Discussion

This study aimed to investigate the physical and psychological impact of SARS‐CoV‐2 infection on cancer patients compared to their co‐living individuals. The results of this study emphasize the complexity of managing cancer patients during the COVID‐19 pandemic, highlighting key differences in physical symptoms, hematological parameters, and psychological outcomes between these two groups. Overall, cancer patients exhibited statistically higher depression and anxiety scores compared to co‐living individuals; however, these differences remained within the minimal severity range and may not necessarily indicate a need for targeted clinical intervention. Instead, these findings highlight the importance of long‐term psychological monitoring and supportive care to ensure mental well‐being in this population.

### 4.1 Baseline and Demographic Characteristics

At baseline, cancer patients had higher depression, anxiety, and fatigue scores compared to co‐living individuals, which can be attributed to the psychological and physical burden of both cancer and its treatment. However, despite this initial difference, both groups demonstrated a significant reduction in psychological distress over time, with no clinically meaningful differences in the degree of improvement.

While certain demographic and clinical factors (such as age [19], cancer type [4], and treatment status [20]) were observed to correlate with psychological scores, these differences did not reach statistical significance in subgroup analyses. This suggests that while these factors may influence the psychological experience of patients, they may not necessarily translate into clinically actionable differences in mental health outcomes.

From a clinical perspective, this finding highlights the importance of broad psychosocial support strategies rather than highly tailored interventions based on specific cancer types or demographic factors. Instead of focusing solely on high‐risk subgroups, healthcare professionals should emphasize general psychological well‐being programs, routine mental health monitoring, and patient‐centered supportive care, as both cancer patients and their co‐living individuals exhibit a tendency toward psychological recovery over time.

### 4.2 Psychological Impact: Depression, Anxiety, and Fatigue

Studies have shown that the global prevalence of anxiety and depression disorders in cancer patients is 31%–46% and 27%–47%, respectively, which may be higher than the prevalence of anxiety disorders (15.15%) and depression (15.97%) in the general population [21, 22]. One of the most striking findings of this study was the significantly higher levels of depression, anxiety, and fatigue among cancer patients compared to co‐living individuals, both at the time of COVID‐19 diagnosis and 1 year later. These psychological outcomes were particularly pronounced in patients with advanced‐stage cancer, as patients with Stage III/IV disease reported significantly higher GAD‐7 and FS‐14 scores compared to those with earlier‐stage disease. These results are consistent with previous research [23] that has demonstrated an increased burden of psychological distress in patients with more advanced cancers, likely due to a combination of factors including greater symptom burden, more intensive treatments, and poorer prognosis [24]. Furthermore, cancer patients undergoing combination therapy exhibited a higher likelihood of experiencing depression, which is consistent with the findings that anticancer treatment is closely associated with patients' anxiety and depression levels [25]. In our study, there were no statistically significant differences in FS‐14, PHQ‐9, or GAD‐7 scores among patients of different ages or different cancer types, which may be due to our small sample size, which included only 72 cancer patients.

Gender differences in psychological outcomes were also evident, with female cancer patients reporting significantly higher depression scores compared to males. This finding is consistent with existing literature on gender differences in psychological responses to illness [26], with women often reporting higher levels of anxiety and depression in the context of chronic illness. This may be explained by a combination of biological, psychological, and sociocultural factors [27]. Hormonal fluctuations (estrogen and progesterone) and neuroinflammatory differences may increase susceptibility to mood disturbances in women [28]. Psychologically, women are more prone to emotional rumination, whereas men tend to adopt problem‐focused coping strategies, which may be protective [29]. Socioculturally, women often face greater caregiving burdens and disruptions in social roles, contributing to higher emotional distress [30]. Additionally, differences in social support networks and gender expectations may further influence mental health outcomes [31]. Understanding these disparities highlights the need for gender‐sensitive psychosocial interventions, such as targeted cognitive‐behavioral therapy (CBT) and stress management programs.

The correlation between lack of COVID‐19 vaccination and increased fatigue in cancer patients further underscores the potential protective effects of vaccination not only against the physical impacts of COVID‐19 but also against the psychological and fatigue‐related sequelae of the disease. Cancer patients who remained unvaccinated were more likely to report severe fatigue, which may be reflective of more severe COVID‐19 symptoms or delayed recovery. This highlights the importance of encouraging vaccination in cancer patients as part of a comprehensive strategy to mitigate both the physical and psychological impacts of the pandemic.

### 4.3 Laboratory Parameters

The significant differences in hematological and immunological parameters between cancer patients and co‐living individuals provide further insight into the vulnerability of cancer patients to infections such as SARS‐CoV‐2. Cancer patients exhibited higher neutrophil counts and lower lymphocyte percentages, likely reflecting both their underlying malignancy and the impact of their treatments, such as chemotherapy, which can compromise immune function [32]. The most prominent effect of SARS‐CoV‐2 on the immune system is lymphocytopenia. Severe lymphopenia contributes to the poor outcomes of some patients with COVID‐19 [33]. The reduction of lymphocytes may be because of direct lysis of lymphocytes by SARS‐CoV‐2, as the lymphocytes express ACE2 on its surface. Lymphocytes apoptosis could also be promoted by the plethora of cytokines such as IL‐6, IL‐10, and TNF [34].

The elevated fibrinogen and D‐dimer levels in cancer patients compared to co‐living individuals are consistent with a hypercoagulable state [35, 36] often seen in both malignancy and COVID‐19, which may explain the increased risk of thromboembolic complications reported in cancer patients infected with SARS‐CoV‐2 [37, 38]. These findings suggest that cancer patients with COVID‐19 may require more aggressive management of coagulopathies to mitigate the risk of adverse outcomes [39]. Increased NLR in cancer patients was also a prominent finding, reflecting a poorer prognosis. Prior research indicated that the risk of death is substantially high in cancer patients with an NLR of 6.5 or higher [37, 38].

The significantly higher complement levels (C3 and C1q) in cancer patients may reflect an exaggerated immune response, which is consistent with reports of increased complement activation in both cancer and COVID‐19 [40, 41]. This could also contribute to the more severe inflammatory responses observed in cancer patients, which may exacerbate both their physical and psychological symptoms during the infection. C1q and C3 were strongly associated with each other and associated with monocyte‐driven classical complement activation [40]. Furthermore, the lack of significant differences in liver function markers such as ALT and AST suggests that liver involvement may not differ significantly between cancer patients and co‐living individuals, despite the well‐documented hepatotoxicity of certain cancer therapies.

### 4.4 Symptom Burden and Psychological Distress

The strong correlations observed between physical symptoms, such as nausea, vomiting, and fatigue, and psychological distress in both cancer patients and co‐living individuals emphasize the complex interplay between physical and mental health. In cancer patients, nausea and vomiting were significantly associated with both higher anxiety (GAD‐7) and depression (PHQ‐9) scores, while fatigue was closely linked to psychological distress, as reflected in the FS‐14 scores. These findings are consistent with the concept of symptom clusters, where multiple symptoms co‐occur and exacerbate one another, leading to a higher overall burden of illness [42]. Addressing these symptom clusters through comprehensive symptom management strategies may be critical in improving the overall well‐being of cancer patients.

The co‐living individuals, while not directly affected by cancer, also exhibited significant associations between physical symptoms and psychological outcomes. For example, muscle pain was significantly associated with higher depression scores (PHQ‐9), and loss of appetite was linked to increased anxiety and fatigue. This suggests that the experience of living with a cancer patient during the pandemic may have had a substantial impact on the physical and mental health of co‐living individuals, possibly due to the stress and uncertainty of the situation, as well as the potential for shared environmental or lifestyle factors.

### 4.5 Clinical Implications and Recommendations

Given the persistent psychological burden observed in cancer patients following SARS‐CoV‐2 infection, there are several strategies that clinicians can adopt to enhance care for this vulnerable group. Vaccine hesitancy remains a challenge in immunocompromised populations, including cancer patients, despite evidence demonstrating the safety and efficacy of COVID‐19 vaccination in this group [43]. To improve vaccine acceptance, healthcare providers should engage in patient‐centered discussions that address misconceptions, emphasize the benefits of vaccination in reducing severe outcomes, and provide clear guidance on booster doses. Additionally, integrating oncology‐led vaccination campaigns, offering vaccines within cancer care facilities, and leveraging peer support groups may enhance patient trust and adherence to vaccination recommendations. The psychological burden of COVID‐19 on cancer patients extends beyond acute illness, often persisting for months post‐infection [44]. To mitigate this impact, regular mental health screening should be incorporated into routine oncology care, using validated tools such as PHQ‐9 and GAD‐7. Evidence suggests that early intervention, including referral to psycho‐oncology services, cognitive behavioral therapy (CBT), and mindfulness‐based interventions, can significantly reduce distress. Furthermore, virtual support groups, caregiver counseling programs, and hospital‐based psychosocial support services may benefit both patients and their co‐living individuals by fostering emotional resilience and reducing the long‐term psychological effects of the pandemic.

### 4.6 Study Limitations

There are several limitations in our study. This was a retrospective and single‐center hospital‐based study. The number of cases remains limited, resulting in a small sample size, and there is no one‐to‐one correspondence between cancer patients and co‐living individuals, and a lack of long‐term survival observation. Our study included several cancers, and the sample size is uneven among the different cancers, and there are differences in cancer stage and treatment. The study population is specific to a certain region, which may limit generalizability.

Overall, this study demonstrates that cancer patients experience significantly higher levels of psychological distress and physical symptoms during COVID‐19 compared to their co‐living counterparts. These findings underscore the need for targeted interventions to address both the psychological and physical aspects of COVID‐19 in cancer patients, as well as the importance of vaccination in mitigating the long‐term impacts of the disease. Future studies should focus on developing and evaluating comprehensive care models that integrate physical and psychological support for both cancer patients and their caregivers, particularly in the context of ongoing pandemic‐related challenges.

## Author Contributions

**Jiayao Liu:** methodology, writing – original draft, formal analysis, conceptualization, writing – review and editing, data curation. **Na Li:** conceptualization, methodology, writing – review and editing, data curation, formal analysis. **Bin Wang:** conceptualization, methodology, writing – review and editing, formal analysis, data curation. **Wujie Zhao:** conceptualization, methodology, writing – review and editing, formal analysis, data curation. **Jie Zhi:** conceptualization, methodology, writing – review and editing, data curation, formal analysis. **Xiaojing Jia:** methodology, conceptualization, writing – review and editing, data curation, formal analysis. **Yitao Jia:** conceptualization, methodology, writing – review and editing, formal analysis, data curation. **Yanqing Tie:** methodology, writing – review and editing, conceptualization, formal analysis, data curation.

## Ethics Statement

The study was approved by the Ethics Committee of Hebei General Hospital. It has been registered in the Chinese Clinical Trial Registry (ID: ChiCTR2300067577).

## Consent

Informed consent was obtained from all individual participants included in the study.

## Conflicts of Interest

The authors declare no conflicts of interest.

## Supporting information


Table S1.

## Data Availability

The datasets generated during and/or analyzed during the current study are available from the corresponding author on reasonable request.
