# Supplementary material for: SARS‐CoV‐2 Infection Aggravates Physical and Mental Health in Cancer Patients Compared to Co‐Living Individuals
Source: Cancer Med. 2025 Mar 25;14(6):e70795. doi: 10.1002/cam4.70795 (PMC11933713; doi:10.1002/cam4.70795)
Supplement: Supplementary file 1 — Table S1. [file CAM4-14-e70795-s001.docx]

Supplementary Material

**Supplementary Table 1**. The differences in FS-14, PHQ-9, GAD-7 scores among cancer patients based on cancer type

|  | Lung carcinoma (n = 11) | Gynecologic malignancies (n = 14) | Hepatobiliary carcinoma (n = 3) | Colorectal carcinoma (n = 11) | Breast carcinoma (n = 12) | Esophageal carcinoma (n = 5) | Head and Neck squamous cell carcinoma (n = 4) | Stomach adenocarcinoma (n = 8) | Others (n = 4) | *P* |
| --- | --- | --- | --- | --- | --- | --- | --- | --- | --- | --- |
| FS-14 Score | 7.36 (3.202) | 5.79 (2.517) | 5.67 (1.528) | 4.73 (3.228) | 4.083 (3.260) | 6.60 (5.595) | 4.75 (3.775) | 6.25 (3.454) | 5.50 (2.380) | 0.482 |
| PHQ-9 Score | 5.00 (4.796) | 4.71 (3.871) | 7.00 (3.61) | 3.73 (3.580) | 3.25 (3.108) | 5.20 (5.070) | 2.50 (4.359) | 4.00 (4.472) | 1.50 (1.732) | 0.659 |
| GAD-7 Score | 4.18 (4.423) | 1.21 (1.929) | 7.67 (7.234) | 2.73 (5.293) | 3.08 (4.814) | 5.20 (4.147) | 0.5 (1.000) | 2.75 (4.773) | 0.75 (0.957) | 0.226 |

PHQ-9: Patient Health Questionnaire-9; GAD-7: General Anxiety Disorder-7; FS-14: 14-item Fatigue scale.
